# A new genetic variant of hereditary apolipoprotein A-I amyloidosis: a case-report followed by discussion of diagnostic challenges and therapeutic options

**DOI:** 10.1186/s12881-019-0755-5

**Published:** 2019-01-21

**Authors:** Myrto Moutafi, Dimitrios C. Ziogas, Spyros Michopoulos, Tina Bagratuni, Vassiliki Vasileiou, Laura Verga, Giampaolo Merlini, Giovanni Palladini, Charis Matsouka, Meletios A. Dimopoulos, Efstathios Kastritis

**Affiliations:** 1 Department of Clinical Therapeutics, Alexandra General Hospital, National and Kapodistrian University of Athens, School of Medicine, 80 Vas Sofias Avenue, 11528 Athens, Greece; 2 Amyloidosis Research and Treatment Center, Foundation Istituto di Ricovero e Cura a Carattere Scientifico (IRCCS) Policlinico San Matteo and Department of Molecular Medicine, University of Pavia, Pavia, Italy

**Keywords:** Rare autosomal disease, ApoAI amyloidosis, Liver, Immune-electron microscopy

## Abstract

**Background:**

Hereditary amyloidosis refers to a wide spectrum of rare diseases with different causative mutations in the genes of various proteins including transthyretin, apolipoprotein AI and AII, gelsolin, lysozyme, cystatin C, fibrinogen Aα-chain, β2-microglobulin, apolipoprotein CII and CIII.

**Case presentation:**

Among hereditary amyloidosis subtypes, we describe here a specific case of Apolipoprotein AI amyloidosis (AApoAI), where the diagnosis began from an almost asymptomatic hepatomegaly followed by the development of primary hypogonadism. Baseline laboratory tests showed increased liver enzymes, while imaging tests revealed a suspected infiltrative liver disease. Patient underwent into liver biopsy and histological examination detected the presence of periodic acid-Schiff (−) and Congo-red (+) amorphous eosinophilic material within normal liver tissue. In the typing of amyloid by immunoelectron microscopy, the liver appeared heavily infiltrated by anti-apoAI (+) amyloid fibrils. Gene sequencing and mutational analysis revealed a single-base mutation at position c.251 T > C resulting in an amino acid substitution from leucine to proline in the mature ApoAI protein. This amino acid change led to lower cleavage and ApoAI deposition into the involved organs. Few years later, our patient remaining without treatment, came with symptoms consistent with primary hypogonadism but testicular involvement with ApoAI deposits could not be proven since the patient refused testicular biopsy. Based on this case, we recap the diagnostic challenges, the clinical manifestations, and the potential treatment options for this indolent hereditary amyloidosis subtype.

**Conclusions:**

This case-report enlarges the clinical picture of ApoAI-driven disease and its complex genetic background and in parallel suggests for a more systematic approach in any case with strong suspicion of hereditary amyloidosis.

## Background

Hereditary amyloidosis includes a group of rare diseases, usually inherited in an autosomal dominant manner and characterized by extracellular deposition of insoluble fibril proteins in target organs, causing disruption of their structure and function [1, 2]. There are many subtypes of hereditary amyloidosis caused by different amyloidogenic mutations in genes coding various proteins that include transthyretin (causing ATTR), apolipoprotein AI and AII (causing AApoAI and AApoAII amyloidosis, respectively), gelsolin (AGel), lysozyme amyloidosis (ALys), cystatin C amyloidosis (ACys), fibrinogen Aα-chain (AFib), β2-microglobulin (Aβ2M), apolipoprotein CII and CIII (causing AApoCII and AApoCIII, respectively) [2, 3]. In the majority of cases no symptoms are presented until adulthood [4] and treatment is focused on addressing symptoms of organ damage and slowing down the production of amyloid when possible.

The *APOA1* gene, located on chromosome 11q23-q24, encodes the primary form of apolipoprotein AI (ApoAI). ApoAI is synthesized as a pre-protein by the liver and the intestine, is cleaved by plasma protease, providing the long mature form and degraded mainly in kidney [5, 6]. ApoAI is the major protein component of high-density lipoprotein particles in the plasma, implicating in cholesterol transport [5]. More than 50 variants of *APOA1* gene are known, of which more than 20 have been associated with hereditary ApoAI amyloidosis (AApoAI) [6]. Each specific mutation in *APOA1* gene results in the deposition of ApoAI in various tissues causing distinct clinical syndromes with different age of onset, pattern of organ involvement, progression rate, and prognosis [6]. Even among individuals with identical variants, the clinical spectrum of disease may be quite heterogeneous. According to the involved target-organs, individuals with AApoAI present clinical manifestations from the liver, kidney, skin and the heart, up to the gonads and adrenal glands [7–10].

In this report, we present the case of a middle-aged male patient with a rare presentation of ApoAI amyloidosis, describing the indolent course of his disease, and discussing the diagnostic challenges and the possible treatment options.

## Case presentation

A 48-year-old man (birthplace and residence: Athens, Greece) with a past medical history of vitiligo, was admitted to our center (Alexandra General Hospital) due to bloating and sense of “fullness” in the right upper quadrant of his abdomen, worsening gradually during the past few weeks before his presentation. No fever or dyspnea was reported and the patient had no signs of chronic liver disease. On physical examination, palpable hepatomegaly was detected but without splenomegaly. The patient reported no symptoms of peripheral or autonomic neuropathy and the neurologic exam was unremarkable.

Liver function tests showed increased alkaline phosphatase (~ 3.5 times upper normal limit, UNL) and gamma-glutaminotransferase levels (~ 8 times UNL) while both alanine aminotransferase (ALT) and aspartate aminotransferase (AST) were only slightly increased (<2xUNL). Serum bilirubin, creatinine and albumin levels were in normal range, no prolongation of clotting time or proteinuria was observed. Following imaging tests, abdominal ultrasound showed a liver span in the mid-clavicular line of 21.5 cm, and in consistence, computed tomography (CT) showed diffuse liver enlargement and a reduction in portal vein diameter. An infiltrative liver disease was suspected.

After the baseline work-up and within the first week of patient admission, liver biopsy was performed and histologic examination revealed the presence of periodic acid-Schiff (PAS) negative amorphous eosinophilic material within normal liver tissue and portal areas. Initial Congo red staining was negative but due to high clinical suspicion for amyloidosis, a second tissue evaluation was performed and was positive for Congo red. However, immunohistochemistry was unclear, since both anti-kappa and anti-lambda as well as anti-AA staining were positive, probably due to absorption. Serum and urine immunofixation were negative and serum free light chains were normal. Cardiac biomarkers (NTproBNP and hsTnT) were within normal range. No evidence of heart involvement was found in cardiac echocardiography.

Liver tissue sample was sent for typing of amyloid to Amyloidosis Research and Treatment Center, Foundation Istituto di Ricovero e Cura a Carattere Scientifico (IRCCS) Policlinico San Matteo and University of Pavia, Italy. By immunoelectron microscopy performed according to the standard already published methodology [11, 12], the liver appeared heavily infiltrated by amyloid fibrils that reacted with anti-apoAI, while no immunoreaction was seen with other antibodies, supporting the diagnosis of AApoAI. To establish the diagnosis of hereditary AApoAI, mutation analysis of the APOA1 gene was performed. By the next generation sequencing, the proband was found to be heterozygous for a single-base substitution at position c.251 T > C, changing the codon *CTG to CCG. This change* resulted in a amino acid substitution, from leucine to proline in the peptidic residue 60 of the mature ApoAI.protein. Sanger sequencing was used for subsequent screening of the proband family members; his siblings (two brothers) and his mother lacked the mutation, but paternal DNA was unavailable.

The patient received no treatment and remained in 6-months follow-up. Two years later he came for his scheduled visit complaining for erectile dysfunction. No other signs or symptoms of autonomic neuropathy were present. Further testing revealed low testosterone levels with concomitant high levels of gonadotrophins, consistent with primary hypogonadism while subsequent semen analysis showed azoospermia. Scrotal ultrasound showed normal size of the testicles. Primary hypogonadism was attributed to testicular involvement, but this hypothesis could not be proven since the patient refused testicular biopsy.

The patient remains in stable very good clinical status for the past 4 years in regular follow-up visits, without evidence of deterioration of his liver or other organ function. His symptoms due to hypogonadism have resolved with testosterone-replacing therapy. The CARE guidelines were followed in reporting this case [13].

## Discussion and conclusions

In this report, we recognized a case of hereditary AApoAI caused by a novel single nucleotide mutation c.251 T > C in the APOA1 gene, described for the first time in a patient of Greek ancestry. This single-base mutation resulted in an amino acid substitution from leucine to proline in the mature ApoAI.protein, leading to lower cleavage and increased deposition of ApoAI into the involved organs. In our case, the ApoAI-driven disease presented initially with liver involvement followed by the development of primary hypogonadism most probably due to the deposition of ApoAI in extracellular testicular tissue. Hypogonadism as a clinical presentation has also been reported in other cases of AApoAI [7–9] and the possibility of testicular or adrenal involvement should be considered in any case of hereditary amyloidosis and gonadal disorders [14, 15].

**Fig. 1:**
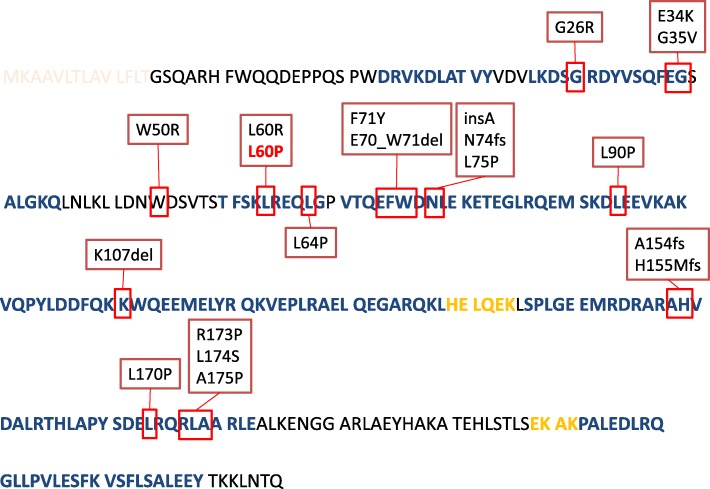
Secondary structure of ApoAI, based on the UNIPROT database (https://www.uniprot.org/uniprot/P02647). Helix is depicted with bold blue letters and turn region with orange bold. The sites of identified mutations, as provided by http://www.amyloidosismutations.com/cdna-aapoai.html are shown with red boxes. The L60P mutation described in the manuscript is also shown in red characters

In general, each specific mutation results in a syndrome with different clinical manifestations and organ involvements [4]. Figure 1 presents the secondary structure of ApoAI, based on the UNIPROT database (https://www.uniprot.org/uniprot/P02647) and pinpoints on this pattern the exact sites of identified mutations (http://www.amyloidosismutations.com/cdna-aapoai.html). For example, another already described mutation in the same *genetic locus* of *APOA1* leads to Leu60Arg amino-acid substitution and has a totally different phenotype [16]. Leu60Pro variant is associated with deposition of amyloid fibrils in the liver without renal involvement while Leu60Arg has precipitated renal dysfunction without liver involvement [16]. This is not surprising since even for identical variants, multiple genetic and environmental factors may contribute to phenotypic variation [17]. Among known mutations, eight (including Gly26Arg, Trp50Arg, Leu60Arg, Leu64Pro, Glu70_Trp72del, Asn74fs, Leu75Pro, Ala154fs) have been associated with renal involvement (with or without liver involvement) [8] and only one with isolated liver involvement (Leu60_Phe71delinsValThr). According to the initial description of a Spanish family with AApoAI amyloidosis caused by heterozygous mutation Leu60_Phe71delinsValThr [18, 19], the diagnosis was first made in two asymptomatic females after undergoing liver biopsies due to abnormal liver function tests during routine screening. Both cases were later found to suffer from primary adrenal insufficiency, which was confirmed by blood tests (high level of ACTH and low level of cortisol) at ages 65 and 66, respectively.

The absence of thorough family history, the lack of recorded symptomatology and of DNA sampling from the paternal side may not allow us to depict the comprehensive inherited figure of the disease but this missing information reflects also the high likehood of loosing such a diagnosis due to relatively indolent disease course with no clear organ dysfunction. The use of immunoelectron microscopy allowed the precise diagnosis and typing of amyloid in this patient. Using as an example our case, the role of amyloid typing with immunoelectron microscopy and mass spectrometry, should be acknowledged and emphasized as critical components of the diagnostic approach [11, 12].

In contrast to AL amyloidosis, in hereditary forms of amyloidosis the elimination of the production of the precursor protein is not usually a realistic target and their management focuses on addressing symptoms in target organs. Some patients with AApoAI amyloidosis may require cardiac, liver or renal transplantation due to severe organ insufficiency over the course of the disease. Liver transplantation (orthotopic transplantation) has been suggested as a therapeutic option, for familial AApoAI [20] when there is extensive liver amyloidosis with significant liver deterioration and/or when there is clinically significant functional impairment caused by amyloidogenic ApoAI in non-transplantable organs, such as peripheral nerves. So far there are no approved therapies, which can eliminate systemic amyloid deposits. A promising approach is to target different circulating components of the amyloid fibrils such as glycosaminoglycans or serum amyloid P-component. In early phase studies, a small-molecule drug, (R)-1-[6-[(R)-2-carboxy-pyrrolidin-1-yl]-6-oxo-hexanoyl]-pyrrolidine-2-carboxylic acid (CPHPC) [21] could deplete circulating serum amyloid P component (SAP), which is found in all amyloid deposits; followed by a single dose of a monoclonal IgG1 anti-SAP antibody [22] it was found to safely trigger clearance of SAP-containing amyloid deposits in liver tissue [23]. All these strategies are under investigation and extensive research is needed before such approaches may become an effective treatment.

In conclusion, this is a case report presenting a previously unknown amyloidogenic single point mutation in APOA1 gene associated with hereditary ApoAI amyloidosis with liver and gonadal involvement. Amyloidosis should be timely considered in individuals presenting with chronic elevation of cholestatic enzymes especially when no other alternative explanation exist. The suspicion of amyloidosis is the most important step in the diagnostic approach; however, advanced technology may be required for the accurate diagnosis and the correct typing of amyloid.

## Abbreviations

AApoAI: ApoAI amyloidosis
AApoAII: amyloidosis by apolipoprotein AII
AapoCII: amyloidosis by apolipoprotein CII
AapoCIII: amyloidosis by apolipoprotein CIII
ACys: cystatin C amyloidosis
AFib: amyloidosis causing by fibrinogen Aα-chain
AGel: amyloidosis by gelsolin;
ALT: alanine aminotransferase
ALys: amyloidosis by lysozyme
ApoAI: Apolipoprotein AI
AST: aspartate aminotransferase
ATTR: amyloidosis causing by transthyretin
Aβ2M: amyloidosis by β2-microglobulin
CPHPC: (R)-1-[6-[(R)-2-carboxy-pyrrolidin-1-yl]-6-oxo-hexanoyl]-pyrrolidine-2-carboxylic acid
CT: computed tomography
HDL: high-density lipoprotein
Leu: `leucine
PAS: periodic acid-Schiff
Pro: proline
SAP: serum amyloid P
UNL: upper normal limit
